# Childhood-onset depression and newly diagnosed chronic diseases after age 65: a large longitudinal cohort study

**DOI:** 10.1186/s12888-025-07494-9

**Published:** 2025-10-27

**Authors:** Zhishuang Li, Zheran Liu, Yaxin Luo, Zhigong Wei, Rendong Huang, Ling He, Ruidan Li, Xiaolin Hu, Xingchen Peng

**Affiliations:** 1 Department of Biotherapy and National Clinical Research Center for Geriatrics, Cancer Center, West China Hospital, Sichuan University, Chengdu, Sichuan China; 2 Department of Biostatistics, Key Laboratory for Health Technology Assessment, Key Laboratory of Public Health Safety of Ministry of Education, School of Public Health, National Commission of Health, Fudan University, Shanghai, China; 3 Department of Epidemiology and Biostatistics, West China Hospital, West China School of Public Health and West China Fourth Hospital/Cancer Center, Sichuan University, Chengdu, China; 4 School of Nursing, Hangzhou Medical College, Hangzhou, Zhejiang China; 5 Department of Nursing, West China Hospital, Sichuan University, Chengdu, China; 6 Sichuan Provincial Key Laboratory of Nuclear Physics and Medical Research, Sichuan University, Chengdu, China

**Keywords:** Childhood-onset depression, Chronic disease, Epidemiology, Primary prevention

## Abstract

**Background:**

The long-term risk of childhood-onset depression (diagnosed depression before age 16) on chronic diseases in the elderly aged 65 years and above was unclear. The aim of present study is to investigate the association between childhood-onset depression and newly diagnosed chronic diseases after age 65.

**Methods:**

12,314 respondents’ data were from fifteen waves (1992–2018) of the Health and Retirement Longitudinal Study (HRS). The association was estimated using the RR (risk ratio) and their 95% CI (confident interval). The chronic disease outcomes included eight chronic diseases (Hypertension, Diabetes, Cancer, Chronic lung disease, Heart problems, Stroke, Emotional/psychiatric problem, Arthritis) newly diagnosed after age 65.

**Results:**

The findings showed that after age 65, the risk of having newly diagnosed chronic diseases was significantly higher in people with childhood-onset depression than without it *(RR 1.31*,* 95% CI 1.12–1.52* *p* < 0.01), and the newly diagnosed of two specific chronic diseases including chronic lung diseases (RR 1.53, 95% CI 1.04–2.16, p-value (Bonferroni) = 0.002) and emotional/psychiatric problem (RR 2.17, 95% CI 1.34–3.31, p-value (Bonferroni) = 0.006) (*P* < 0.05/8 is significant) after age 65 were found to be significantly associated with childhood-onset depression.

**Conclusions:**

Childhood-onset depression would increase the risk of newly diagnosed chronic diseases, specifically chronic lung diseases, and emotional/psychiatric problem in the elderly. Future clinical research and health policy should be targeted at the root cause to explore the causal pathway between childhood-onset depression and later-life chronic diseases.

**Supplementary Information:**

The online version contains supplementary material available at 10.1186/s12888-025-07494-9.

## Introduction

Depression is a global mental health disorder and a leading cause of disability [1], which is associated with both physical and psychiatric conditions. The bad influence of depression is still expanding and deepening, such as among the healthcare workers infected with COVID-19, those with lower mental health suffered more damage [2]. However, the sequential relationship between childhood-onset depression and chronic disease in later age is not clear, and little is known about the impact of childhood-onset depression.

Childhood-onset depression (depression diagnosed before age 16) is a real and unique clinical symptom, which is a serious and easily masked health condition that tends to deteriorate into serious chronic comorbidity and be recurrent, over time to increase disability. Furthermore, according to the report of the World Health Organization, childhood-onset depression is thought to be the fourth leading cause of suicide in people aged between 15 and 29 [3].

Without early diagnosis and treatments, it would exacerbate the complexity of the disease and increase economic burden [4].Thus, it is particularly important to understand the potential and long-term risk of childhood-onset depression to the occurrence and development of chronic diseases.

Although the exact definition of the elderly age group is controversial, the World Health Organization (WHO) defines an elderly person as 65 years old or older [5]. It has been estimated that the number of people aged 60 and over will increase to 1.2 billion in 2025 and subsequently to two billion in 2050 [6]. These demographic transitions necessarily require a shift in global focus to cater to the preventive healthcare of the elderly population [7]. Furthermore, this elderly population already have an overburdened healthcare delivery system than the younger population [1]. Therefore, it is important to focus on the health of the elderly (older than 65 years), through exploring the risk factors and promoting early prevention methods, to improve occurrence and outcomes of chronic diseases as well as diseases survivors for the elderly [8].

Although the recognition of the importance of childhood-onset depression is increasing among individuals and communities, unfortunately, as an important part and sensitive episode of depression, childhood-onset depression still lacks the related analysis about its long-term effects and association with the newly diagnosed chronic diseases in old age. As far as we know, a population-based longitudinal study is still necessary to provide evidence for this link. We propose to examine the relationship between them using a large longitudinal cohort in an American population. The outcome would support the suggestion that early identification of childhood-onset depression will help to reduce the potential risk of chronic diseases in the elderly population, decrease medical costs as well as improve quality of life in later age.

## Methods

### Study design

The analysis was based on the HRS, which was supported by the National Institute on Aging (NIA U01AG009740) and the Social Security Administration and was conducted by the University of Michigan [9]. HRS collected nationally representative samples comprising 42,233 individual respondents aged 45 years conducted in each biennial wave from 1992 (wave 1) to 2020 (wave 15).

**Fig. 1:**
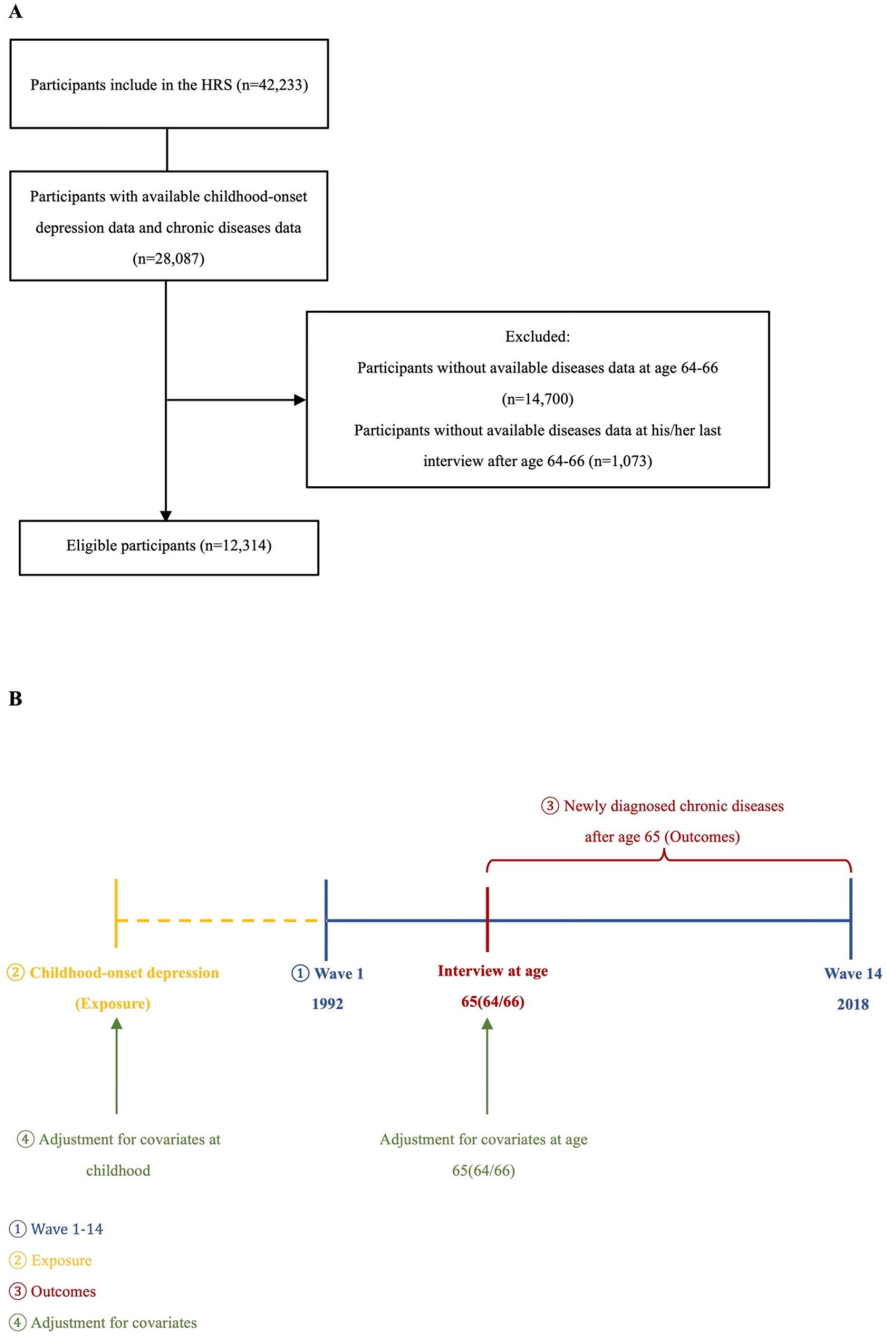
**A** The flow chart of the study population. **B** The timeline of analyses. HRS: Health and Retirement Longitudinal Study

Participants in the longitudinal cohort were identified from retrospective reports on HRS participants in each biennial wave during 1992–2018 (*n* = 42,233). Participants were excluded if they: (1) lack the information on childhood-onset depression (*n* = 14,146); (2) lack the information on chronic disease situations at age 64–66 (*n* = 14,700); (2) lack the information on chronic disease situations in his/her last interview after age 65 (*n* = 1,073). A total of 12,314 participants were included (shown in Fig. 1A).

### Ascertainment of exposure and outcomes

The data about whether participants had childhood-onset depression is from self-reported information in childhood questionnaires at 2016 (wave 13). In the questionaries, participants were asked “Before you were 16 years old, did you have any of the following childhood diseases: Depression?”, The answer is yes, no, DK (Don’t Know); NA (Not Ascertained), RF (Refused) [9]. The latter two are defined as not known in our present study.

Disease-related outcomes data were obtained from the HRS questionnaire. In the questionaries, participants were asked “Has a doctor ever told you that you have the following specified condition? “. Whether a special chronic disease is newly diagnosed after the age 65 depends on the participants’ responses in the last interview after age 65 and in the interview at the age 65(64/66) (the interviews were conducted in each biennial wave). Eight chronic diseases were included: (1) Hypertension (high blood pressure or hypertension); (2) Diabetes (diabetes or high blood sugar); (3) Cancer (cancer or a malignant tumor of any kind except skin cancer); (4) Chronic lung disease (chronic lung disease except asthma such as chronic bronchitis or emphysema); (5) Heart problems (heart attack, coronary heart disease, angina, congestive heart failure, or other heart problems); (6) Stroke (stroke or transient ischemic attack (TIA)); (7) Emotional/psychiatric problem (emotional, nervous, or psychiatric problems); (8) Arthritis (arthritis or rheumatism) [9]. The first outcome of the study was defined as whether having any newly diagnosed chronic diseases (excluding emotional/psychiatric problem because of its qualitative difference) after age 65 (Yes/No). Other outcomes were defined as whether having eight specific newly diagnosed chronic diseases.

For analyzing any newly diagnosed specific outcome, the individual who had been diagnosed the corresponding chronic disease before age 65 would be excluded (shown in Fig. 1B). The selection of chronic diseases takes into account the information provided by the database and referred to the outcomes’ selection of the relevant study [10].

### Ascertainment of covariates

Information on confounders is divided into three parts: (1) Demographic information: gender (male or female), marital status (married, widowed, single/other), educational level (illiterate, middle school and lower, high school and higher), birth year; (2) Childhood background (before age 16): rate health as child (fair/poor, excellent/good); parents/guardians smoke (yes/no); childhood smoking (yes/no); childhood drugs/alcohol using (yes/no); social economic station (SES) (poor, average, pretty well, varied); family problems caused by parents’ drinking and drugs (yes/no); physically abused by parents (yes/no). (3) Health condition and behavior: BMI (body mass index) (< = 24.9, 25–29.9.9, >=30), self-reported health status (fair/poor, excellent/good), drinking alcohol now (yes/no), smoking now (yes/no) and number of chronic diseases at age 65 as health status at age 65. We employed a complete case analysis for the covariates. Subjects with missing data were excluded from the analysis. Additionally, a sensitivity analysis using multiple imputation was conducted to assess the potential impact of missing data on the results.

### Statistical analyses

All analyses were conducted with R Version 4.2.1, and a two-sided *p* < 0.05 was set as statistically significant. The associations between childhood-onset depression and whether having any newly diagnosed chronic diseases after age 65 as well as whether having eight specific newly diagnosed chronic diseases after age 65 were evaluated by the generalized linear model with log link for Poisson regression to estimate risk ratios (RRs) and 95% CIs. As the hypothesis of this study is on overall chronic diseases, we used the Bonferroni method to adjust the significance level to α = 0.00625 (0.05/8 (number of test)) when analyzing the eight specific newly diagnosed chronic diseases. Only adjusted p-values less than 0.00625 were considered statistically significant.

Four models were established according to the degree of adjustment. For the selection and inclusion of covariables, we followed the experience. Through including, in turn, non-modifiable demographic variables, life-related variables in childhood, and health and life-related variables at age 65, we further revealed the relationship between exposure to outcomes. Model 1 was a univariate model for childhood-onset depression. Model 2 was additionally adjusted for (1) Demographic information (excluding potential mediating factors). Model 3 was further adjusted for (2) Childhood background (before age 16). Model 4 was further adjusted for (3) Health conditions and behavior at age 65. In addition, the number of been diagnosed chronic diseases at age 65 was additional adjusted.

### Subgroup analyses

We conducted subgroup analyses to evaluate potential effect modifications by Demographic information; Childhood background (before age 16); Health condition and behavior. For the analysis of “any newly diagnosed chronic disease,” individuals with any pre-existing chronic disease diagnosed before age 65 were excluded. For disease-specific analyses (i.e., cancer, chronic lung disease, and emotional/psychiatric problems), only individuals without a pre-existing diagnosis of the corresponding disease before age 65 were included. Interaction terms between childhood-onset depression and each subgroup variable were introduced into the multivariable Poisson regression models. The likelihood ratio test was used to assess statistical significance of interaction effects.

### Sensitivity analysis

To address potential bias introduced by missing data in covariates, we conducted a sensitivity analysis using multiple imputation. Under the assumption that data were missing at random (MAR), we implemented the multiple imputation by chained equations (MICE) approach using the R ‘mice’ package.

## Results

### Characteristics of participants with and without childhood-onset depression

Participant characteristics are summarized in Table 1 and Table S1. Among 12,314 eligible participants, 12,025 (97.6%) participants were without childhood-onset depression and 289 (2.34%) participants were self-reported diagnosed as childhood-onset depression. Compared with participants without childhood-onset depression, those with childhood-onset depression were more likely to be female, not be married, suffer from more chronic diseases, report fair health at age 65 and during childhood, have a poor family financial situation, experience traumatic events and use drug/alcohol.

**Table 1:** The baseline characteristics of participants stratified by childhood-onset depression

	Without childhood-onset depression	With childhood-onset depression	Overall	*P* value
N	12,025	289	12,314	
Birth year (mean (sd))	1933.01 (29.93)	1947.70 (30.12)	1933.87 (30.16)	0.085
Gender				< 0.001
male	5139 (42.7%)	86 (29.8%)	5225 (42.4%)	
female	6886 (57.3%)	203 (70.2%)	7089 (57.6%)	
Educational level				0.691
Illiterate	77 (0.6%)	3 (1.0%)	80 (0.6%)	
Middle school and lower	6517 (54.2%)	154 (53.3%)	6671 (54.2%)	
High school and higher	5403 (44.9%)	131 (45.3%)	5534 (44.9%)	
Missing	28 (0.2%)	1 (0.3%)	29 (0.2%)	
Marital status				< 0.001
Married	8075 (67.2%)	146 (50.5%)	8221 (66.8%)	
Single/other	2488 (20.7%)	107 (37.0%)	2595 (21.1%)	
Widowed	1451 (12.1%)	36 (12.5%)	1487 (12.1%)	
Missing	11 (0.1%)	0 (0%)	11 (0.1%)	
Self-reported health status				< 0.001
Excellent/good	9131 (75.9%)	162 (56.1%)	9293 (75.5%)	
Fair/Poor	2885 (24.0%)	126 (43.6%)	3011 (24.5%)	
Missing	9 (0.1%)	1 (0.3%)	10 (0.1%)	
BMI				0.307
<=24.9	3192 (26.5%)	86 (29.8%)	3278 (26.6%)	
25–29.9.9	4611 (38.3%)	99 (34.3%)	4710 (38.2%)	
>=30	4048 (33.7%)	99 (34.3%)	4147 (33.7%)	
Missing	174 (1.4%)	5 (1.7%)	179 (1.5%)	
Drinking alcohol now				0.092
No	5657 (47.0%)	151 (52.2%)	5808 (47.2%)	
Yes	6364 (52.9%)	138 (47.8%)	6502 (52.8%)	
Missing	4 (0.0%)	0 (0%)	4 (0.0%)	
Smoking now				0.166
No	5003 (41.6%)	106 (36.7%)	5109 (41.5%)	
Yes	6942 (57.7%)	176 (60.9%)	7118 (57.8%)	
Missing	80 (0.7%)	7 (2.4%)	87 (0.7%)	
Number of chronic diseases at baseline				< 0.001
0	1844 (15.3%)	15 (5.2%)	1859 (15.1%)	
1–4	9466 (78.7%)	225 (77.9%)	9691 (78.7%)	
5–8	621 (5.2%)	42 (14.5%)	663 (5.4%)	
Missing	94 (0.8%)	7 (2.4%)	101 (0.8%)	
SES				< 0.001
Pretty Well	803 (6.7%)	23 (8.0%)	826 (6.7%)	
Average	7402 (61.6%)	124 (42.9%)	7526 (61.1%)	
Poor	3679 (30.6%)	139 (48.1%)	3818 (31.0%)	
Varied	114 (0.9%)	2 (0.7%)	116 (0.9%)	
Missing	27 (0.2%)	1 (0.3%)	28 (0.2%)	

*Note* N: the number of observations. BMI: body mass index. SES: socioeconomic status. Data are n (%)

### Risk of newly diagnosed chronic diseases between participants with childhood-onset depression and those without it

For analyzing the association between childhood-onset depression and having any newly diagnosed chronic diseases after age 65, the individual who had been diagnosed any chronic disease before age 65 would be excluded. Table 2 indicated the association between childhood-onset depression and newly diagnosed chronic diseases after age 65. By adjusting more covariates from model 1 to model 4, we found the association is significant in models 2, 3, and 4. In the fourth model, participants with childhood-onset depression were more likely to have newly diagnosed chronic diseases after age 65 (model 4: RR 1.31, 95%CI 1.12–1.52, *P* < 0.001), which suggested that childhood-onset depression might have long-term effects on the aggravation of chronic diseases condition in older persons. Note. N: the number of observations. RR: Risk ratio

**Table 2:** Unadjusted models and adjusted models estimated the association between childhood-onset depression and having any newly diagnosed chronic disease after age 65

	*N*	RR (95%CI)	*P* value
Model 1^a^	12,213	0.91 (0.77–1.08)	0.32
Model 2^b^	12,176	1.17 (1.01–1.34)	0.039
Model 3^c^	10,714	1.22 (1.06–1.41)	< 0.01
Model 4^d^	10,499	1.31 (1.12–1.52)	< 0.01

(a) model 1: Univariate model

b. model 2 additionally adjusted for gender, 609 marital status, educational level, and birth year

(c) model 3 additionally adjusted for the rate health as child, parents/guardians smoke, childhood smoking, childhood drugs/alcohol, SES: socioeconomic status, family problems caused by parents’ drinking and drugs, physical abused by parents.

(d) model 4 additionally adjusted for BMI, self-reported health status, drinking alcohol now, smoking now, the number of been diagnosed chronic diseases at age 65

For analyzing the association between childhood-onset depression and any newly diagnosed specific chronic diseases after age 65, the individual who had been diagnosed the corresponding chronic disease before age 65 would be excluded. Table 2 further indicated that childhood-onset depression was significantly associated with increased risk of newly diagnosed cancer (RR 1.68, 95%CI 1.36–2.10, p-value (Bonferroni) = 0.004) in model 1, chronic lung disease (RR 1.53, 95%CI 1.04–2.16, p-value (Bonferroni) = 0.002) in model 4 and emotional/psychiatric problem (RR 2.17, 95%CI 1.34–3.31, p-value (Bonferroni) = 0.006) in model 4 after age 65 in the model 4, which further suggested that childhood-onset depression might increased risk of having these chronic diseases even participants have never been diagnosed with those.

To avoid adjusting for potential mediators, Model 2 in Tables 2 and 3 presents results adjusted only for baseline confounders, including sex, educational level, marital status, and year of birth. The findings demonstrate that further adjustment for potential intermediate factors only influenced the magnitude of the risk ratios, without altering the statistical significance of the associations.

**Table 3:** Unadjusted models and adjusted models estimated the association between childhood-onset depression and specific newly diagnosed chronic disease after age 65

Outcome	Number	Number of CD^e^	Estimate	*p*-value	*p*-value (Bonferroni)
Model 1^a^					
Hypertension	5520	115	0.87 (0.62–1.18)	0.394	1
Diabetes	9669	228	1.04 (0.73–1.43)	0.814	1
Cancer	10,869	244	1.68 (1.36–2.10)	< 0.001	0.004*
Chronic lung disease	11,284	235	1.82 (1.28–2.50)	< 0.001	0.003*
Heart problems	9946	209	0.96 (0.70–1.29)	0.811	1
Stroke	11,582	251	1.11 (0.71–1.64)	0.620	1
Arthritis	5189	80	0.91 (0.60–1.32)	0.634	1
Emotional/psychiatric problem	10,392	116	2.62 (1.68–3.87)	< 0.001	< 0.001*
Model 2 ^b^					
Hypertension	5505	114	0.90 (0.64–1.23)	0.539	1
Diabetes	9644	228	1.09 (0.76–1.50)	0.617	1
Cancer	10,833	243	1.54 (1.17–2.02)	0.005	0.041
Chronic lung disease	11,246	234	1.83 (1.29–2.52)	< 0.001	0.004*
Heart problems	9916	208	1.05 (0.76–1.40)	0.762	1
Stroke	11,545	250	1.16 (0.74–1.71)	0.479	1
Arthritis	5171	79	0.95 (0.62–1.37)	0.789	1
Emotional/psychiatric problem	10,360	116	2.58 (1.66–3.82)	< 0.001	< 0.001*
Model 3 ^c^					
Hypertension	4939	106	0.94 (0.66–1.31)	0.742	1
Diabetes	8605	206	1.04 (0.71–1.46)	0.842	1
Cancer	9513	215	1.41 (0.98–1.97)	0.211	1
Chronic lung disease	9911	212	1.57 (1.08–2.19)	< 0.001	0.0013*
Heart problems	8751	189	1.04 (0.75–1.41)	0.803	1
Stroke	10,202	223	1.20 (0.76–1.80)	0.406	1
Arthritis	4493	67	0.97 (0.63–1.43)	0.892	1
Emotional/psychiatric problem	9167	108	2.35 (1.49–3.50)	< 0.001	0.001*
Model 4 ^d^					
Hypertension	4837	100	0.95 (0.65–1.33)	0.778	1
Diabetes	8436	192	1.11 (0.76–1.57)	0.558	1
Cancer	9329	200	1.23 (0.75–1.74)	0.637	1
Chronic lung disease	9706	198	1.53 (1.04–2.16)	< 0.001	0.002*
Heart problems	8561	175	1.01 (0.71–1.38)	0.967	1
Stroke	9994	207	1.23 (0.78–1.84)	0.348	1
Arthritis	4412	61	0.94 (0.59–1.42)	0.780	1
Emotional/psychiatric problem	8984	98	2.17 (1.34–3.31)	< 0.001	0.006*

*Note*. N: the number of observations. *RR* Risk ratio. *p-value (Bonferroni)* Bonferroni method was used to adjust the significance level to α = 0.00625 (0.05/8 (number of test)) when analyzing the eight specific newly diagnosed chronic diseases. Only adjusted p-values less than 0.00625 (marked as *) were considered statistically significant. *CD* Childhood-onset depression; SES: socioeconomic status

(a) model 1: univariate model

(b) model 2 additionally adjusted for gender, marital status, educational level and birth year

(c) model 3 additionally adjusted for the rate health as child, parents/guardians smoke, childhood smoking, childhood drugs/alcohol, SES, family problems caused by parents’ drinking and drugs, physical abused by parents

(d) model 4 additionally adjusted for BMI, self-reported health status, drinking alcohol now, smoking now, and the number of been diagnosed chronic diseases at age 65

(e) The number of those with childhood-onset depression in each analysis

### Subgroup analysis

**Fig. 2:**
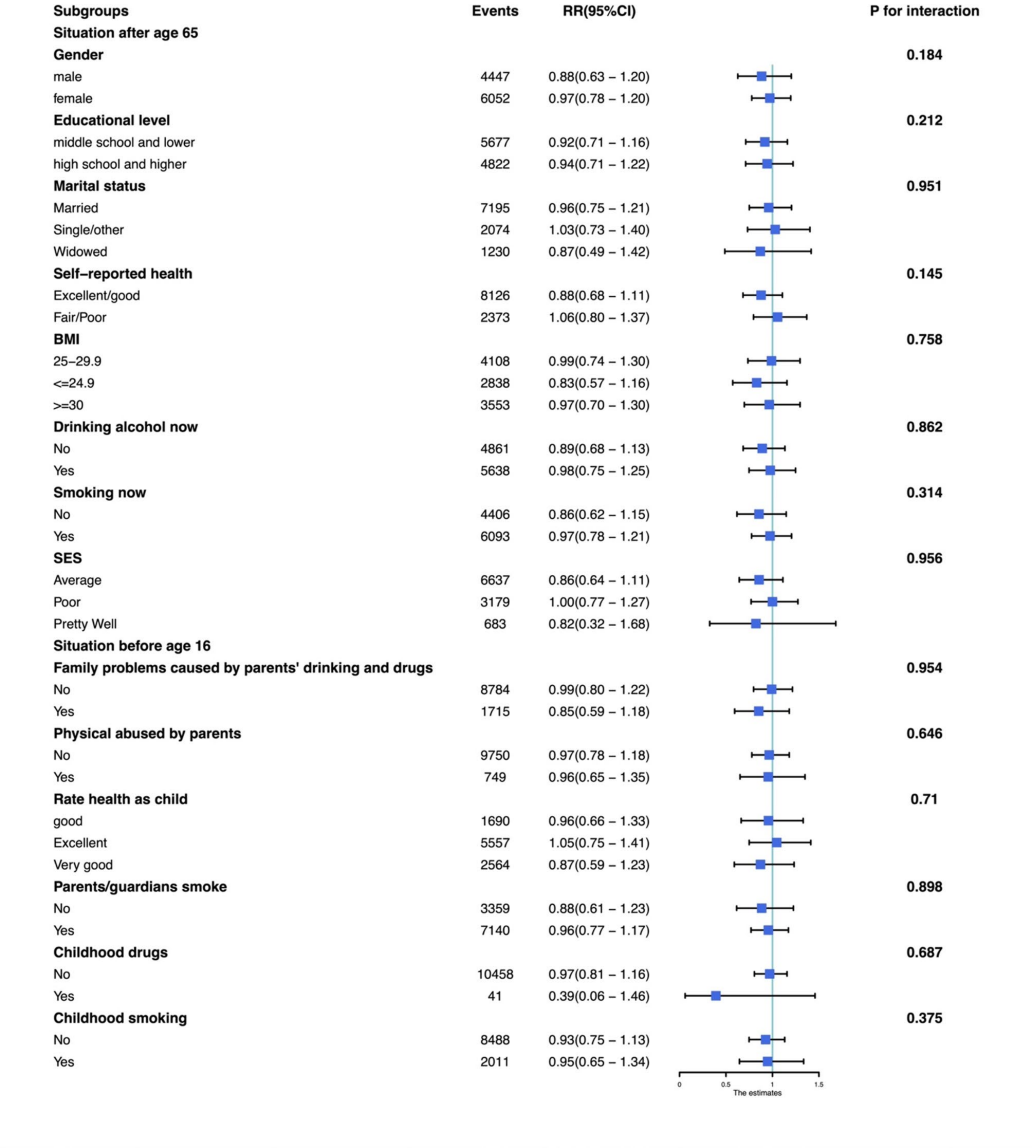
Subgroups analysis about the association between childhood-onset depression and whether having any chronic diseases emerging after age 65 Note. BMI: body mass index. SES: socioeconomic status

According to our results, childhood-onset depression is associated with an increased risk of newly diagnosed chronic diseases. Subgroup analyses were used to examine whether different characteristics could modify these associations (shown in Fig. 2), the individual who had been diagnosed any chronic disease before age 65 would be excluded. We found that the increased risk of having any newly diagnosed chronic diseases in people with childhood-onset depression was independent of all covariates. In a subgroup analysis of three disease outcomes (shown in Figure S1-S3), the individual who had been diagnosed the corresponding chronic disease before age 65 would be excluded. We found that the increased risk of newly diagnosed cancer disease in people with childhood-onset depression was independent of all variables except self-reported health status at age 65 (*P* = 0.009). Among people who reported good health at age 65, the adverse effects of childhood-onset depression were not significant, while childhood-onset depression increased risk of newly diagnosed cancer in people with poor health (RR 2 0.3, 95%CI 1.21–3.1). For chronic lung disease and emotional/psychiatric problem, the associations between childhood-onset depression and increased risk of newly diagnosed disease were independent of all of the variables mentioned above. In addition, the consistency of results between the complete-case analysis and the multiple imputation analysis supports the robustness of our findings to missing data assumptions.

## Discussion

In the present longitudinal study, we divided the participants according to their exposure to childhood-onset depression and followed them from the age of 65 to their last interview to obtain the outcomes of the newly diagnosed chronic diseases. By adjusting covariates in four models, our results showed that participants with childhood-onset depression have more risk of having newly diagnosed chronic diseases after age 65, particularly newly diagnosed cancer, chronic lung disease and emotional/psychiatric problem. In the conclusion, childhood-onset depression might increase risk of newly diagnosed chronic diseases after age 65, of which the impact might be long-term for older persons across the entire life cycle.

Previous evidence has shown the existence of an association between the prevalence of depression and chronic diseases. In Gabilondo’s study in Spain, the comorbidity of the major depressive episode might deteriorate chronic physical conditions [11]. The association was also found in other four chronic diseases: angina, arthritis, asthma, and diabetes in the World Health Surveys [12]. Half a million prospective cohorts and meta-analysis had found that increased prevalence of chronic diseases among people with depression according to the evidence [13].

However, these studies were mostly about adulthood depression and single chronic diseases, which neglected the potential long-term damage of childhood-onset depression and its effects on the chronic disease condition of the elderly. In addition, limiting the cross-sectional or short-term observational study, the causal relationship between both and the potential long-term effects of childhood-onset depression lack longitudinal cohort research based on high-quality sampling and especially.

Multiple inquired information and wide range of waves in HRS helped us to attain information about childhood-onset depression and outcomes accurately. By applying a single independent variate of childhood-onset depression and associating it with newly diagnosed chronic diseases after age 65, we established a longitudinal cohort and found the long-term adverse effects of childhood-onset depression on health in later age. Although there has been an association between childhood-onset depression and chronic diseases such as obesity [14], the long-term effects of childhood-onset depression on newly diagnosed chronic diseases after age 65 were first identified in our study.

Our study provides robust evidence that childhood-onset depression is significantly associated with an increased risk of developing specific chronic diseases in later life, even after rigorous adjustment for socio-demographic, early-life, and health-related covariates. Notably, the associations with chronic lung diseases and emotional/psychiatric problems remained statistically significant across all models after Bonferroni correction, underscoring the enduring and independent nature of these links.

Childhood-onset depression demonstrated distinct associations with the risk of specific chronic diseases in later life. A significantly elevated risk of cancer was observed in minimally adjusted models (Model 1), though this association was attenuated after accounting for health behaviors and baseline health status, suggesting a mediating role of lifestyle factors and physiological dysregulation such as chronic inflammation and HPA axis activation [15, 16]. In contrast, the association with chronic lung diseases remained robust and statistically significant across all models, indicating strong independent links potentially involving stress-related respiratory dysfunction, systemic inflammation, and health behaviors [17, 18]. Most notably, childhood-onset depression was consistently and strongly associated with emotional/psychiatric problems in adulthood, even after full adjustment, supporting theories of psychological “scarring” and neurobiological vulnerability that predispose individuals to lifelong mental health challenges [19, 20]. These findings collectively highlight the critical need for integrated prevention and long-term monitoring strategies that address both mental and physical health trajectories in individuals with a history of early-life depression.

From a clinical perspective, these findings advocate for integrated care models that address both mental and physical health trajectories in individuals with a history of childhood-onset depression. Regular screening for chronic conditions—particularly respiratory and mental health disorders—should be considered in this population. Furthermore, public health efforts should focus on early mental health interventions and health behavior promotion starting in childhood to alter long-term health trajectories.

Moreover, Further analyses are warranted but we can predict that the explanation of this potential pathway may be the individual characteristics precipitating the onset of chronic disease and after the development of childhood-onset depression. There have been some researchers finding a way to how childhood-onset depression influences the development of chronic diseases. Firstly, baseline depressive symptoms were significantly associated with physical inactivity as well as lower health status in a prospective community-based study [21]. Secondly, Evidence has shown that people with childhood-onset depression are most likely to have adverse health behavior such as using Tobacco [22], high cortisol levels, which are the risk factors for chronic diseases like cardiovascular disease (CVD) and chronic lung diseases in the long term [23, 24]. Thirdly, research has revealed that the association between childhood-onset depression and metabolic syndrome was significantly among women (but not men) younger than 40 years [ 25].

The study supports that childhood-onset depression had a long-term influence on newly diagnosed chronic disease after age 65 and several outcomes of this study strengthen the evidence. First, HRS is a national panel survey of individuals over the age of 50 and their spouses. Its multiple data provide us with the opportunity to investigate the many different aspects related to the aging of the United States. population and to explore more potential covariates to reveal true associations. Secondly, we use the outcome-wide approach to analyze the potential associations. Full-outcome studies are advocated in population studies as opposed to full-exposure studies because this method has the advantages of low investigation bias, good comparability of effective scale and high effectiveness [26].

Several limitations should be considered when interpreting our findings. First, survival bias is a concern, as individuals who died prior to the initiation of the HRS were necessarily excluded from the study, potentially limiting the generalizability of our results. Second, both exposure and outcome assessments relied on self-reported questionnaire data, which are subject to recall bias. To partially address variations in recall related to age at reporting and differences in follow-up time, we adjusted for birth year in all models. Third, although adjusting for potential mediators in our fully specified models may have introduced overcontrol bias, our Model 1 (unadjusted) and Model 2 (adjusted for baseline confounders only) indicated that such adjustments generally affected the magnitude of risk estimates rather than their statistical significance—with the exception of cancer, which was attenuated after additional adjustments. Fourth, although the study is longitudinal, the absence of continuous follow-up from childhood to late adulthood means that important intermediate factors (e.g., adulthood socioeconomic status, healthcare utilization, and health trajectories) were not fully captured. For example, individuals with childhood-onset depression often experience higher rates of healthcare use and familial financial strain, which may influence chronic disease risk through pathways not fully measured in this study [27–29]. Although we adjusted for several early-life and adulthood confounders (“SES”, “Rate health as a child”), residual confounding due to these unmeasured intermediate factors remains possible. Therefore, our results should not be interpreted as establishing causality, and further studies using lifelong longitudinal cohorts are needed to better delineate the causal pathways linking childhood-onset depression to later chronic health outcomes. Fifth, survival bias may have led to an underestimation of the association between childhood-onset depression and later-life adverse health outcomes, as the analysis only included individuals who survived into old age and participated in the HRS. Sixth, the sample size of the exposed group (i.e., individuals with childhood-onset depression, *n* = 289) was relatively small, which limits the statistical power for detecting smaller effects and the precision of our estimates, particularly in the subgroup analyses.

By using a wide outcome approach in a large population-based longitudinal cohort study in the United States, this study, after adjusting for multiple covariates, reveals that childhood-onset depression is associated with the risk of newly diagnosed chronic diseases after age 65, particularly with chronic lung disease, cancer, and emotional/psychiatric problem, and has long-term harm to later age. Depression plays an important role in the onset, development, and prognosis of chronic diseases. At the clinical level, the correlation of this study indicates that timely diagnosis and treatment of childhood-onset depression may greatly reduce the occurrence and development of chronic diseases in old age, avoid deterioration of older people’s health, and reduce the burden of chronic diseases on the older persons in society.

Therefore, it is necessary to enhance early identification of childhood-onset depression. At the epidemiological level, the results indicate that childhood-onset depression may represent an independent risk factor for newly diagnosed chronic diseases in older adults. These findings underscore the importance of placing greater emphasis on the prevention of childhood-onset depression in future public health efforts.

## Supplementary Information

Supplementary material 1

## Data Availability

The datasets analyzed during the current study are available in the Health Retirement Study (HRS, [https://hrsonline.isr.umich.edu](https:/hrsonline.isr.umich.edu)). The HRS questionnaire link is [https://www.rand.org/well-being/social-and-behavioral-policy/centers/aging/dataprod/hrs-data.html](https:/www.rand.org/well-being/social-and-behavioral-policy/centers/aging/dataprod/hrs-data.html).
