# Supplementary material for: Childhood-onset depression and newly diagnosed chronic diseases after age 65: a large longitudinal cohort study
Source: BMC Psychiatry. 2025 Oct 27;25:1025. doi: 10.1186/s12888-025-07494-9 (PMC12560582; doi:10.1186/s12888-025-07494-9)
Supplement: Supplementary file 1 — Supplementary material 1 [file 12888_2025_7494_MOESM1_ESM.docx]

SUPPLEMENTAL MATERIAL

Table S1. The childhood baseline characteristics of participants stratified by childhood-onset depression.

|  | Without childhood-onset depression | With childhood-onset depression | Overall | P value |
| --- | --- | --- | --- | --- |
| N (%) | 12025 | 289 | 12314 |  |
| SES |  |  |  | <0.001 |
| Pretty Well | 803 (6.7%) | 23 (8.0%) | 826 (6.7%) |  |
| Average | 7402 (61.6%) | 124 (42.9%) | 7526 (61.1%) |  |
| Poor | 3679 (30.6%) | 139 (48.1%) | 3818 (31.0%) |  |
| Varied | 114 (0.9%) | 2 (0.7%) | 116 (0.9%) |  |
| Missing | 27 (0.2%) | 1 (0.3%) | 28 (0.2%) |  |
| Family problems caused by parents’ drinking and drugs |  |  |  | <0.001 |
| Yes | 1697 (14.1%) | 89 (30.8%) | 1786 (14.5%) |  |
| No | 8959 (74.5%) | 172 (59.5%) | 9131 (74.2%) |  |
| Missing | 1369 (11.4%) | 28 (9.7%) | 1397 (11.3%) |  |
| Physical abuse by parents |  |  |  | <0.001 |
| Yes | 704 (5.9%) | 77 (26.6%) | 781 (6.3%) |  |
| No | 9965 (82.9%) | 184 (63.7%) | 10149 (82.4%) |  |
| Missing | 1356 (11.3%) | 28 (9.7%) | 1384 (11.2%) |  |
| Rate health as child |  |  |  | <0.001 |
| Excellent | 6356 (52.9%) | 79 (27.3%) | 6435 (52.3%) |  |
| Very good | 2921 (24.3%) | 72 (24.9%) | 2993 (24.3%) |  |
| Good | 1984 (16.5%) | 69 (23.9%) | 2053 (16.7%) |  |
| Fair | 615 (5.1%) | 43 (14.9%) | 658 (5.3%) |  |
| Poor | 149 (1.2%) | 26 (9.0%) | 175 (1.4%) |  |
| Parents/guardians smoke |  |  |  | 0.224 |
| Yes | 8077 (67.2%) | 208 (72.0%) | 8285 (67.3%) |  |
| No | 3908 (32.5%) | 80 (27.7%) | 3988 (32.4%) |  |
| Childhood drugs/alcohol |  |  |  | <0.001 |
| Yes | 36 (0.3%) | 16 (5.5%) | 52 (0.4%) |  |
| No | 11989 (99.7%) | 273 (94.5%) | 12262 (99.6%) |  |
| Childhood smoking |  |  |  | 0.129 |
| Yes | 2264 (18.8%) | 68 (23.5%) | 2332 (18.9%) |  |
| Outcomes |  |  |  |  |
| Hypertension |  |  |  | **0.749** |
| 0 | 3402 (28.3%) | 78 (27.0%) | 3480 (28.3%) |  |
| 1 | 2003 (16.7%) | 37 (12.8%) | 2040 (16.6%) |  |
| Missing | 6620 (55.1%) | 174 (60.2%) | 6794 (55.2%) |  |
| Diabetes |  |  |  | **0.916** |
| 0 | 8049 (66.9%) | 193 (66.8%) | 8242 (66.9%) |  |
| 1 | 1392 (11.6%) | 35 (12.1%) | 1427 (11.6%) |  |
| Missing | 2584 (21.5%) | 61 (21.1%) | 2645 (21.5%) |  |
| Cancer |  |  |  | **0.873** |
| 0 | 9261 (77.0%) | 210 (72.7%) | 9471 (76.9%) |  |
| 1 | 1364 (11.3%) | 34 (11.8%) | 1398 (11.4%) |  |
| Missing | 1400 (11.6%) | 45 (15.6%) | 1445 (11.7%) |  |
| Chronic lung disease |  |  |  | **0.005** |
| 0 | 10118 (84.1%) | 199 (68.9%) | 10317 (83.8%) |  |
| 1 | 931 (7.7%) | 36 (12.5%) | 967 (7.9%) |  |
| Missing | 976 (8.1%) | 54 (18.7%) | 1030 (8.4%) |  |
| Heart problems |  |  |  | **0.318** |
| 0 | 7706 (64.1%) | 167 (57.8%) | 7873 (63.9%) |  |
| 1 | 2031 (16.9%) | 42 (14.5%) | 2073 (16.8%) |  |
| Missing | 2288 (19.0%) | 80 (27.7%) | 2368 (19.2%) |  |
| Stroke |  |  |  | **0.899** |
| 0 | 10396 (86.5%) | 228 (78.9%) | 10624 (86.3%) |  |
| 1 | 935 (7.8%) | 23 (8.0%) | 958 (7.8%) |  |
| Missing | 694 (5.8%) | 38 (13.1%) | 732 (5.9%) |  |
| Emotional/psychiatric problem |  |  |  | **<0.001** |
| 0 | 9499 (79.0%) | 93 (32.2%) | 9592 (77.9%) |  |
| 1 | 777 (6.5%) | 23 (8.0%) | 800 (6.5%) |  |
| Missing | 1749 (14.5%) | 173 (59.9%) | 1922 (15.6%) |  |
| Arthritis |  |  |  | **0.108** |
| 0 | 3352 (27.9%) | 55 (19.0%) | 3407 (27.7%) |  |
| 1 | 1757 (14.6%) | 25 (8.7%) | 1782 (14.5%) |  |
| Missing | 6916 (57.5%) | 209 (72.3%) | 7125 (57.9%) |  |

Note. N: the number of observations. SES: social economic status.

Figure S1. Subgroups analysis about the association between childhood-onset depression and the newly diagnosed cancer after age 65. RR: Risk ratio, SES: socioeconomic status.

Figure S2. Subgroups analysis about the association between childhood-onset depression and newly diagnosed chronic lung diseases after age 65. RR: Risk ratio, SES: socioeconomic status.

Figure S3. Subgroups analysis about the association between childhood-onset depression and newly diagnosed emotional/psychiatric problem after age 65. RR: Risk ratio, SES: socioeconomic status.

Table S2. Unadjusted models and adjusted models estimated the association between childhood-onset depression and specific newly diagnosed chronic disease after age 65 (Missing values are filled with multiple imputation).

| **Outcome** | **Number** | **Number of CD^e^** | **Estimate** | **p-value** | **p-value (Bonferroni)** |
| --- | --- | --- | --- | --- | --- |
| **Model 1^a^** |  |  |  |  |  |
| **Hypertension** | 5520 | 115 | 0.87 (0.62-1.18) | 0.394 | 1 |
| **Diabetes** | 9669 | 228 | 1.04 (0.73-1.43) | 0.814 | 1 |
| **Cancer** | 10869 | 244 | 1.68 (1.36-2.10) | <0.001 | 0.004* |
| **Chronic lung disease** | 11284 | 235 | 1.82 (1.28-2.50) | <0.001 | 0.003* |
| **Heart problems** | 9946 | 209 | 0.96 (0.70-1.29) | 0.811 | 1 |
| **Stroke** | 11582 | 251 | 1.11 (0.71-1.64) | 0.620 | 1 |
| **Arthritis** | 5189 | 80 | 0.91 (0.60-1.32) | 0.634 | 1 |
| **Emotional/psychiatric problem** | 10392 | 116 | 2.62 (1.68-3.87) | <0.001 | <0.001* |
| **Model 2 ^b^** |  |  |  |  |  |
| **Hypertension** | 5520 | 115 | 0.90 (0.64-1.23) | 0.537 | 1 |
| **Diabetes** | 9669 | 228 | 1.09 (0.76-1.50) | 0.613 | 1 |
| **Cancer** | 10869 | 244 | 1.54 (1.17-2.02) | 0.005 | 0.042 |
| **Chronic lung disease** | 11284 | 235 | 1.83 (1.29-2.51) | <0.001 | 0.003 |
| **Heart problems** | 9946 | 209 | 1.05 (0.76-1.41) | 0.760 | 1 |
| **Stroke** | 11582 | 251 | 1.16 (0.74-1.71) | 0.480 | 1 |
| **Arthritis** | 5189 | 80 | 0.95 (0.62-1.37) | 0.784 | 1 |
| **Emotional/psychiatric problem** | 10392 | 116 | 2.61 (1.67-3.85) | <0.001 | <0.001* |
| **Model 3 ^c^** |  |  |  |  |  |
| **Hypertension** | 5520 | 115 | 0.94 (0.66-1.30) | 0.731 | 1 |
| **Diabetes** | 9669 | 228 | 1.04 (0.71-1.46) | 0.842 | 1 |
| **Cancer** | 10869 | 244 | 1.41 (0.98-1.97) | 0.210 | 1 |
| **Chronic lung disease** | 11284 | 235 | 1.56 (1.08-2.19) | <0.001 | 0.0013* |
| **Heart problems** | 9946 | 209 | 1.04 (0.75-1.41) | 0.802 | 1 |
| **Stroke** | 11582 | 251 | 1.20 (0.76-1.80) | 0.405 | 1 |
| **Arthritis** | 5189 | 80 | 0.97 (0.63-1.43) | 0.891 | 1 |
| **Emotional/psychiatric problem** | 10392 | 116 | 2.37 (1.51-3.53) | <0.001 | <0.001* |
| **Model 4 ^d^** |  |  |  |  |  |
| **Hypertension** | 5520 | 115 | 0.95 (0.65-1.33) | 0.771 | 1 |
| **Diabetes** | 9669 | 228 | 1.11 (0.76-1.57) | 0.554 | 1 |
| **Cancer** | 10869 | 244 | 1.23 (0.75-1.74) | 0.018 | 0.144 |
| **Chronic lung disease** | 11284 | 235 | 1.53 (1.04-2.17) | <0.001 | 0.003* |
| **Heart problems** | 9946 | 209 | 1.01 (0.71-1.38) | 0.957 | 1 |
| **Stroke** | 11582 | 251 | 1.23 (0.78-1.84) | 0.349 | 1 |
| **Arthritis** | 5189 | 80 | 0.94 (0.59-1.41) | 0.777 | 1 |
| **Emotional/psychiatric problem** | 10392 | 116 | 2.19 (1.35-3.35) | 0.001 | 0.005* |

Note. N: the number of observations. RR: Risk ratio. **p-value (Bonferroni):** Bonferroni method was used to adjust the significance level to α = 0.00625 (0.05/8 (number of test)) when analyzing the eight specific newly diagnosed chronic diseases. Only adjusted p-values less than 0.00625 (marked as *) were considered statistically significant. CD: Childhood-onset depression.

a. model 1: univariate model. b. model 2 additionally adjusted for gender, marital status, educational level and birth year. c. model 3 additionally adjusted for the rate health as child, parents/guardians smoke, childhood smoking, childhood drugs/alcohol, SES: socioeconomic status, family problems caused by parents’ drinking and drugs, physical abused by parents. d. model 4 additionally adjusted for BMI, self-reported health status, drinking alcohol now, smoking now, and the number of been diagnosed chronic diseases at age 65. e. The number of those with childhood-onset depression in each analysis.
